# Glaucoma and Corneal Transplant Procedures

**DOI:** 10.1155/2012/576394

**Published:** 2012-01-18

**Authors:** Ammar M. Al-Mahmood, Samar A. Al-Swailem, Deepak P. Edward

**Affiliations:** ^1^Department of Ophthalmology, Ministry of Health, Manama 12, Bahrain; ^2^Division of Anterior Segment, King Khaled Eye Specialists Hospital, Riyadh 11462, Saudi Arabia; ^3^Wilmer Eye Institute, Johns Hopkins University School of Medicine, Baltimore, MD 21287, USA

## Abstract

Glaucoma after corneal transplantation is a leading cause of ocular morbidity after penetrating keratoplasty. The incidence reported is highly variable and a number of etiologic factors have been identified. A number of treatment options are available; surgical intervention for IOP control is associated with a high incidence of graft failure. IOP elevation is less frequently seen following deep anterior lamellar keratoplasty. Descemet's striping-automated endothelial keratoplasty is also associated with postprocedure intraocular pressure elevation and secondary glaucoma and presents unique surgical challenges in patients with preexisting glaucoma surgeries. Glaucoma exists in up to three-quarters of patients who undergo keratoprosthesis surgery and the management if often challenging. The aim of this paper is to highlight the incidence, etiology, and management of glaucoma following different corneal transplant procedures. It also focuses on the challenges in the diagnosis of glaucoma and intraocular pressure monitoring in this group of patients.

## 1. Introduction

The primary goal after corneal transplantation is reestablishment of visual acuity for the patient. Corneal transplant surgery has evolved markedly in the past decades from a process of simple replacement of the whole corneal thickness as in penetrating keratoplasty to include deep lamellar keratoplasty (DALK), Descemet's striping-automated endothelial keratoplasty (DSAEK), and keratoprosthesis (KPro). Achieving good visual acuity requires a clear graft and low and regular corneal astigmatism but could be limited by glaucoma and retinal pathology. Unfortunately, the onset and/or progression of glaucoma in patients undergoing transplantation remains a challenge with difficulties faced in the diagnosis and management of these patients. The aim of this paper is to highlight the incidence, etiology, and management of glaucoma following different corneal transplant procedures. It also focuses on the complexity on diagnosing glaucoma and monitoring intraocular pressure in this group of patients. A brief overview over procedures that alters the cornea including corneal refractive surgery and corneal collagen crosslinking (CXL) is also included.

## 2. Glaucoma and Penetrating Keratoplasty

Glaucoma is a serious complication after PKP because of its high incidence and severity and the challenges associated with its diagnosis and treatment [1]. Postkeratoplasty glaucoma represents the second leading cause of graft failure after graft rejection [2–5].

### 2.1. Incidence of Glaucoma Following PKP

A number of studies have reported on the incidence of glaucoma following PKP. França et al. [6] studied incidence of glaucoma in 228 patients who underwent PKP. Forty-nine patients (21.5%) developed glaucoma. In another study by Karadag et al. [7] that included 749 eyes in 729 patients, which underwent PKP, intraocular pressure (IOP) increased in the early postoperative period in 41 (5.5%) eyes and chronically elevated IOP was reported in 124 (16.6%) eyes. The average period between surgery and the first IOP elevation was 5.0 ± 6.5 months for all eyes. The mean IOP value of eyes that developed glaucoma after PKP was 27.9 ± 5.8 mm Hg. Al-Mohaimeed et al. [8] studied prevalence for escalation of glaucoma therapy after PKP in 715 consecutive eyes of 678 patients that underwent PKP. Escalation of glaucoma therapy occurred in 89 (12.4%) eyes of 715 PKP procedures during a mean followup of 32.2 months, out of which 29 eyes had preexisting glaucoma. Wagoner et al. [9] reported worsening of preexisting glaucoma in 15.5% of 66 adult patients who underwent primary optical PKP. Studies by Goldberg et al. [10], Kirkness and Ficker [11], Polack [12], and Simmons et al. [13] also reported a low incidence of secondary open-angle ocular hypertension after PKP in keratoconus and Fuchs' dystrophy. The rate of glaucoma occurrence in keratoconus and Fuchs' dystrophy was similar. In summary of the literature, the incidence of secondary glaucoma after PKP is highly variable, ranging from 10% to 42% that depended on the surgical indication of PKP and the complexity of surgery [10–17].

### 2.2. Etiology and Risk Factors of Glaucoma Following PKP

The pathophysiology of post-PKP glaucoma is multifactorial and may be related to distortion of the angle with collapse of the trabecular meshwork, suturing technique, postoperative inflammation, use of corticosteroids, peripheral anterior synechiae (PAS) formation, and preexisting glaucoma [18]. Olson and Kaufman [19], using a mathematical model, proposed that the elevated IOP following PKP in an aphakic patient might be the result of angle distortion secondary to a compressed tissue in the angle. Edema and inflammation after surgery lead to further compromise in the trabecular meshwork function, and the situation is further aggravated by angle distortion. Factors that contribute to angle distortion include tight suturing, long bites, larger trephine sizes, smaller recipient corneal diameter, and increased peripheral corneal thickness.

Zimmerman et al. [20] proposed that the mechanical collapse of the trabecular meshwork in aphakic grafts was the main problem leading to glaucoma. They postulated that the trabeculum needs posterior fixation offered by the ciliary body-lens support system and an anterior support offered by the descemet's membrane. In aphakia, the posterior support is relaxed with the removal of the lens. After PKP, Descemet's membrane is incised, which leads to a relaxation of the anterior support. Both these factors lead to a partial trabecular collapse and obstruction of aqueous outflow. It would be interesting to evaluate some of these hypothesis related to trabecular alteration as a cause of glaucoma following PKP using newer anterior segment imaging techniques that can visualize the trabecular outflow pathways.

Retained viscoelastic material is another important cause for increased IOP in the early postoperative period, especially with the use of cohesive viscoelastics and its combination with chondroitin sulfate used during PKP.

The leading cause for late post-PKP glaucoma, however, is synechial angle closure with the degree of synechial closure strongly correlated with the need for glaucoma surgery [21]. A floppy atrophic iris may also lead to a higher incidence of PAS formation, which can be prevented by iris suturing or iridoplasty [22].

The incidence of postoperative IOP elevation is associated with surgical indication. The lowest incidence of IOP rise was reported in the patients with keratoconus [10, 13, 14]. Bullous keratopathy, graft rejection, history of glaucoma, and trauma were reported to be high-risk factors for IOP elevation following PKP [11–13]. Wagoner et al. [9] reported that eyes with corneal edema were more likely to develop than those with stromal scaring (*P* < 0.001).

Preoperative glaucoma was identified as a major risk factor for post-PKP glaucoma in many studies [3, 5, 10, 14, 15]. Karadag et al. [7] included 32 patients with preoperative glaucoma who had medical or surgical treatment before surgery. The incidence of post-PKP glaucoma was 59.4% in eyes with preexisting glaucoma in contrast to 14.6% in cases without such a history (*P* = 0.0001). Also, the control of IOP significantly worsened in cases with preoperative glaucoma. The preoperative diagnoses of the patients who developed glaucoma was graft rejection in 27 (21.7%) patients, pseudophakic bullous keratopathy in 24 (19.3%) patients, aphakic bullous keratopathy in 14 (11.2%) patients, corneal scar in 14 (11.2%) patients, vascularized scar in nine (7.2%) patients, trauma in five (4%) patients, keratoconus in nine (7.2%) patients, corneal dystrophy in five (4%) patients, corneal abscess in four (3.2%) patients, graft thinning in four (3.2%) patients, graft abscess in three (2.4%) patients, scar formation secondary to herpetic keratitis in two (1.6%) patients, and band keratopathy in one (0.8%) patient.

PAS formation preoperatively or as a consequence of a preceding intraocular surgery was significantly associated with the development of postoperative glaucoma [11, 13, 23]. Some studies found that aphakic and pseudophakic eyes in the presence of PAS had a greater tendency to develop post PKP glaucoma when compared with phakic eyes [12–14]. Other studies found no difference between aphakic and pseudophakic eyes but reported a higher incidence of post-PKP glaucoma in pseudophakic and aphakic eyes compared with phakic [7].

Some authors reported an increased in the relative risk associated with post-PKP glaucoma following combined surgical procedure with PKP [11, 13]. Seitz et al. [24] assessed the impact of the trephination method and simultaneous cataract surgery on the early and long-term IOP after PKP in eyes without previous surgery and glaucoma in patients with keratoconus and Fuchs' dystrophy. An IOP > 21 mm Hg and/or application of topical antiglaucoma medication was documented in 9% of patients where excimer laser-assisted trephination was performed versus 15% of control patients that underwent traditional trephination of the corneal button (*P* = 0.32), in 15% of Fuchs' dystrophy versus 11% of keratoconus cases (*P* = 0.41) and in 11% of PKP only versus 15% of triple-procedure cases (*P* = 0.68). The IOP elevation started an average of 3.7 ± 2.8 months (1 week to 9 months) after PKP. They concluded that there was no detectable impact from the trephination method, the diagnosis, or simultaneous cataract surgery. This was supported by other studies that did not report a statistically significant difference in IOP following PKP between patients who underwent a combined procedure and those who did not [6, 14].

The presence of ocular inflammation before in the pre- or postoperative period is an important risk factor for post-PKP glaucoma [6, 18, 25].

Prolonged use of topical steroids after PKP makes these patients more vulnerable to complications including IOP elevation. Steroid-induced IOP elevation is one of the important causes of late-onset postkeratoplasty glaucoma [26]. Pramanik et al. [27] reported steroid-induced glaucoma in 4 (3.6%) of 112 eyes of patients with keratoconus after PKP with a mean followup of 13.8 years. Erdurmus et al. [28] evaluated the frequency of steroid-induced IOP elevation and/or glaucoma in patients with keratoconus compared with patients with Fuchs' endothelial dystrophy after PKP. A total of 100 patients with keratoconus and 58 patients with Fuchs dystrophy were included in this study. The overall frequency of steroid-induced IOP elevation after PKP was 73% in the keratoconus group and 60.3% in the Fuchs dystrophy group. The frequency of IOP elevation of at least 5 or 10 mm Hg over the preoperative baseline was 72% and 24% in keratoconus group and 56.9% and 20.7% in the Fuchs dystrophy group, respectively. The frequency of IOP elevation ≥22 or ≥30 mm Hg was 22% and 6% in the keratoconus group and 29.3% and 1.7% in the Fuchs dystrophy group, respectively. Most IOP measurements were done by Goldmann's applanation tonometry unless irregularity of the mires precluded accurate readings. In these cases and during the first week after surgery a Tono-Pen (Medtronic Solan Mentor, Norwell, MA, USA) was used. No difference between the groups in terms of frequency of IOP elevation was observed (*P* > 0.05 for all). Fan et al. [29] reported a 53% elevation of IOP in 57 eyes that occurred mostly 3–6 months following keratoplasty. In summary, IOP elevation related to steroid use is fairly common and requires careful monitoring.

Interestingly concomitant glaucoma surgery was found to be significant risk factor for graft failure, but simultaneous cataract surgery was not [30].

Knowledge of the risk factors may help one to take appropriate measures to limit the occurrence of glaucoma following PKP and increase the chances of success of the corneal graft.

### 2.3. IOP Measurement and Assessment of Glaucoma Damage Following PKP

Following PKP, changes in corneal thickness, postoperative astigmatism, and refractive changes often preclude reliable postoperative assessment of IOP, disc, and visual field.

IOP in the early postoperative period, when the corneal surface is irregular, can be measured with the Mackay-Marg electronic applanation tonometer [31], the pneumatic applanation tonometer, the Tono-Pen, or the dynamic contour tonometer (DCT). These instruments appear to measure IOP independent of the corneal thickness within certain ranges of IOP [18]. If the corneal graft surface is smooth with an intact epithelium and regular mires can be obtained, then Goldmann's applanation (GAT) can be used to measure the IOP. The accuracy of applanation tonometry is reduced in certain situations, such as corneal edema, scars, blood staining, or any condition that thickens or alters the corneal elasticity. Corneal epithelial edema and stromal edema predispose to inaccurately low readings, whereas pressure measurements taken over a corneal scar will be falsely high [32]. One of the challenges of measuring or evaluating IOP preoperatively can be the status of the cornea. The presence of corneal scarring or edema may make more difficult to accurately measure IOP in the preoperative period, and the degree of suture-induced astigmatism following PKP may also make the measurement of IOP challenging. Studies reporting IOP changes from baseline in eyes with marked alterations in the preoperative state must be interpreted cautiously. The use of a pneumatonometer and frequency-doubling perimetry were reported as helpful supplemental methods to detect early glaucomatous damage in patients after PKP independent of postoperative topographic changes of the cornea [33].

### 2.4. Management of Glaucoma Following PKP

#### 2.4.1. Medical Management

The use of topical medications to control IOP is still the first-line treatment of post-PKP glaucoma. When using topical drugs to lower the IOP, one has to keep in mind the side effects that are peculiar to them in the setting of post-PKP glaucoma. Beta-adrenergic blockers can lead to superficial punctate keratopathy, exacerbation of dry eye, and corneal anesthesia. Alpha-2-adrenergic agonist drugs can lead to allergic periocular reactions, superficial punctate keratopathy, and dry eyes [34]. The use of miotics in this setting is discouraged, because they promote breakdown of blood aqueous barrier, thus stimulating graft rejection and increasing the risk of retinal detachment, particularly in aphakes. The prolonged use of topical carbonic anhydrase inhibitors can lead to graft decompensation in the presence of borderline corneal endothelial status [35]. Finally, the prostaglandin analogs should also be used with caution as they may lead to uveitis, cystoid macular edema (CME) in aphakia and pseudophakia, and reactivation of herpes simplex keratitis in patients grafted with a previous history of healed herpetic keratitis [36, 37]. The use of adrenergic agents like epinephrine, dipivefrin, is also discouraged in the modern-day management of these patients because of their potential corneal epithelial toxicity, exacerbation of CME in aphakes, pseudophakes, and promotion of conjunctival inflammation thereby making future surgical intervention all the more difficult [18].

In cases of steroid responsive glaucoma, the dose of steroid drops may be tapered to the minimum required. Alternatively, steroids such as Prednisolone or dexamethasone that are potent IOP elevating agents can be replaced by steroids that have a decreased tendency to increase IOP such as topical fluorometholone, loteprednol, and rimexolone. To prevent graft rejection, Cyclosporine A 0.5–2.0% topical drops four times daily can be applied in conjunction with weaker topical steroids to prevent steroid-related ocular hypertension [38].

#### 2.4.2. Surgical Management

**Trabeculectomy**

Conventional trabeculectomy is usually not effective due to dense perilimbal scarring resulting in an increased risk of failure. The failure rate is further increased in aphakic eyes where a vitrectomy is required to prevent vitreous from blocking the trabeculectomy ostium. Antimetabolites must be used in these patients to inhibit the fibroblastic response [39]. The reported success rate of IOP control with mitomycin trabeculectomy in patients with post-PKP glaucoma is 67–91% after a mean followup of  23 ± 13  months [40, 41].

**Glaucoma Drainage Devices (GDDs)**

**Figure 1:**
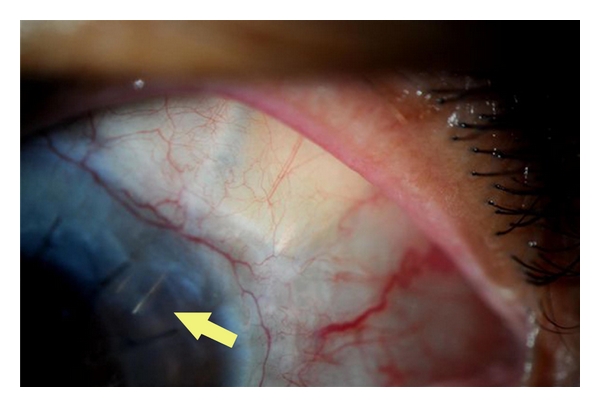
Glaucoma drainage device (Ahmed tube) combined with PKP.

The Use of GDDs Appears to Control Glaucoma in a High Percentage of Patients in All Published Series (71–96%, with an Average of 84.8%) (Figure 1). However, Placement of a GDD Appears to be Associated with a High Incidence of Graft Failure in the Range of 10–51% (Average 36.2%) after a Followup of 8 to 74 Months [42–44]. The Risk of Graft Rejection May be Increased after GDD Surgery, Because the Drainage Tube May Provide a Conduit for Retrograde Passage of Inflammatory Cells Into the AC. The Risk of Graft Rejection is Similar with Valved or Nonvalved GDD's [18]. The Authors Believe That Implantation of GDDs Behind the Iris May Play a Role to Reduce the Flow of Inflammatory Cells Into AC and Therefore Reduce the Rate of Endothelial Cell Loss or Graft Rejection (Figure 4).

**Cyclodestructive Procedures**

Methods of cyclodestruction used to control IOP elevation after PKP include cyclocryotherapy, Nd:YAG laser cyclophotocoagulation (CPC), diode laser CPC, transpupillary argon laser photocoagulation, and endoscopic CPC. Cyclocryotherapy, transscleral CPC with diode, or krypton laser are the various procedures that can be performed on patients with intractable post-PKP glaucoma. CPC is a widely adopted procedure, because it is noninvasive and can be done as a low-cost outpatient procedure [18]. It is especially useful in eyes which develop intractable elevation of IOP in the early postoperative period after PKP. In addition it is a useful modality to control IOP in eyes that have undergone multiple ocular procedures and severe conjunctival scarring and eyes with poor visual potential.

In summary, despite the great relation between glaucoma and PKP, the mechanisms appear to be multifactorial. However, many mechanisms that have been proposed are not completely understood. The lack of prospective studies in this regard leads to variation in the reported rate of glaucoma following PKP. Prospective studies are required to evaluate the mechanism and incidence of glaucoma following PKP.

## 3. Glaucoma and Deep Anterior Lamellar Keratoplasty

**Figure 2:**
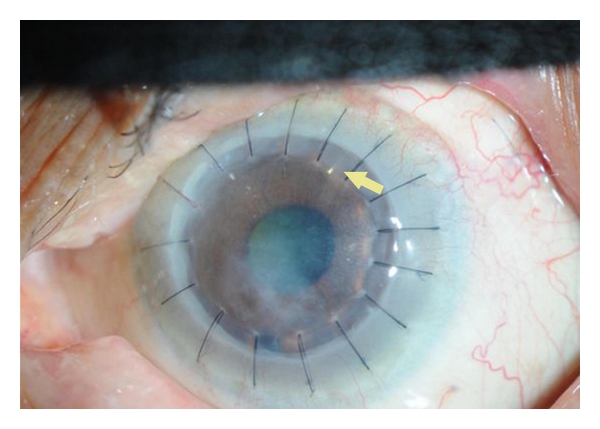
Glaucoma drainage device (Ahmed tube) combined with DALK.

The incidence of glaucoma following DALK was reported to range from zero to 9% after an average followup of 16.0 ± 10.3 months. None of the eyes in the reported series which consisted of a small number of eyes required glaucoma surgery to control IOP. Escape of air through trabecular meshwork when performing big bubble technique can lead to transient elevation of IOP. If air got entrapped behind the iris, then pupillary block glaucoma can develop. If control of IOP elevation is required, the management of glaucoma following DALK is the same as that following PKP [45, 46] (Figure 2).

## 4. Glaucoma and Descemet's Striping-Automated Endothelial Keratoplasty

DSAEK has become a popular technique for the treatment of corneal endothelial dysfunction. Even after corneal edema has resolved, the corneal thickness after DSAEK remarkably increases when compared to a normal cornea, because of the addition of the thickness of the donor graft. The average corneal thickness of the cornea following DSAEK is reported to be 690 ± 77 *μ*m [47].

### 4.1. Incidence of Glaucoma Following DSAEK

In multiple reports, the incidence of induced glaucoma has been reported to be from zero to 18% [48–52]. Vajaranant et al. [53] reported a relatively high incidence of IOP elevation after DSAEK in 35% of patients with no prior glaucoma, 45% of patients with prior glaucoma, and 43% of patients with prior glaucoma with preexisting glaucoma surgery. The overall incidence appears to be lower, less severe, and with better outcomes than that reported with PKP.

### 4.2. Etiology of Glaucoma Following DSAEK

In the early postoperative period, pupillary block from air behind the pupil may occur. This is an uncommon cause of IOP elevation but often leads to significant complications such as graft failure and chronic glaucoma [54]. In the later postoperative period, the development of PAS and prolonged steroid use are important causes. Another mechanism of glaucoma after DSAEK could be distortion of the angle leading to increased IOP. However, this seems less likely as the incision is much smaller than that with traditional PKP. Inflammatory glaucoma is also possible but less likely.

### 4.3. IOP Measurement Following DSAEK

 In clinical use, GAT remains a gold standard for measurement of IOP. It is, however, calibrated for a mean corneal thickness of 520 *μ*m. The increased corneal thickness in patients with DSAEK, however, does not affect IOP measurements by GAT as reported by Vajaranant et al. [55] and others [56]. Additional dynamic contour tonometry (DCT) and pneumotonometry which may measure IOP independent of corneal thickness, curvature, and hydration within certain ranges of IOP may be useful methods to measure IOP following DSAEK [57–60].

### 4.4. Management of Glaucoma Following DSAEK

#### 4.4.1. Medical Management

Vajaranant et al. [55] reported that glaucoma medications were started during the first year after DSAEK in 18% of patients without preexisting glaucoma and were increased in 33% of patients with preexisting glaucoma. Most patients managed well medically with increase in their glaucoma medications and/or tapering of steroids or switch to less potent steroids.

#### 4.4.2. Surgical Management

**Figure 3:**
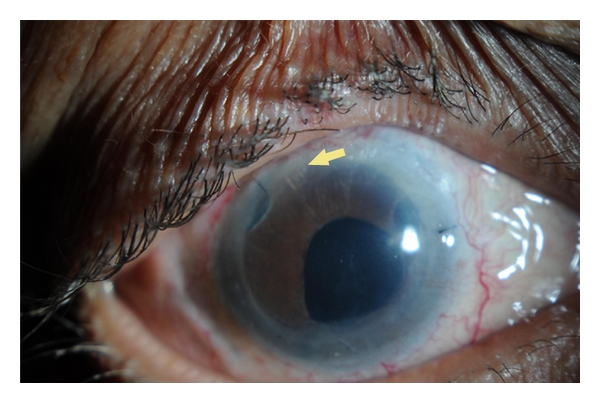
Glaucoma drainage device (Ahmed tube) combined with DSAEK (Courtesy of Dr. Jose Morales).

**Figure 4:**
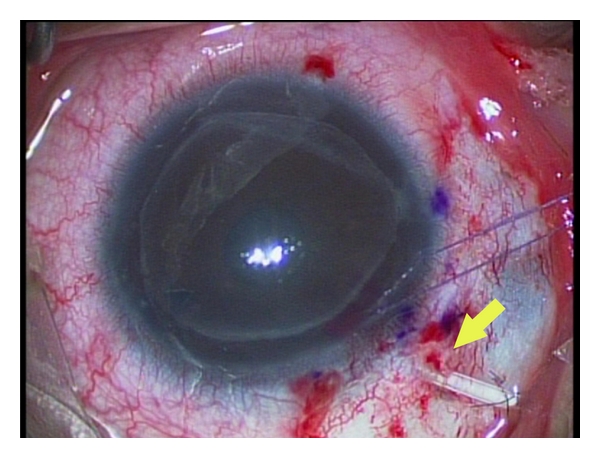
Glaucoma drainage device (Ahmed tube) implanted behind the iris in a case of DSAEK following pesudophakic bullous keratopathy (courtesy of Dr. Jose Morales).

Vajaranant et al. [55] reported 0.3% patients without preexisting glaucoma (1 out of 315) and 8% patients with preexisting glaucoma (7 out of 85) that required glaucoma surgery following DSAEK. The risk of surgical intervention was greater in patients with preexisting glaucoma or in patients with previous glaucoma surgery. The options of trabeculectomy, GDDs, or cyclodestructive procedures are all valid. Usually the approach is similar to that of PKP. Challenges that might be faced include working with a slightly shallow anterior chamber that is not uniform in depth because of edge of graft. Although the optimal tube location for DSAEK patients is not known, primary tube insertion into the posterior chamber may become the preferred option. The tube lumen of a GDD device can potentially be blocked if it impinges against the thick edge of the corneal graft. Using a short tube in anterior chamber placed tangentially into the anterior chamber angle is also advisable (Figure 3). Graft dislocation is not considered an issue if GDDs being placed after and not during DSAEK.

DSAEK is a relatively new procedure, and the short-term incidence of glaucoma and treatment outcomes seem promising. Studies describing long-term IOP and IOP treatment-related outcomes will be important in the future.

## 5. Glaucoma and Keratoprosthesis

Glaucoma is common and permanent blinding sequel of KPro surgery. Several types of KPro are in use; Boston KPro is the most commonly used in the USA, whereas the osteo-odontokeratoprosthesis is used more frequently elsewhere.

### 5.1. Incidence of Glaucoma Following KPro

The prevalence of glaucoma in patients undergoing KPro placement ranges from 36 to 76%. De novo glaucoma has been reported to occur in 2–28% of patients after KPro implantation [61–64]. Preexisting glaucoma in eyes that undergo keratoprosthesis is not uncommon.

### 5.2. Etiology of Glaucoma Following KPro

As most KPro recipients have undergone penetrating keratoplasty, some degree of synechial angle closure usually already exists and progressive angle closure is presumed to be a causative factor. Crowding the anterior segment by the KPro's large backplate that is placed in close proximity to the iris can compromise the angle. Leaving a patient aphakic or removing the iris to eliminate scaffolding can distort and collapse the trabecular meshwork [59]. Topical medications may also play a role in the progression of glaucoma in KPro recipients as the use of topical steroids for prolonged periods following KPro implantation can lead to steroid induced glaucoma [65].

### 5.3. IOP Measurement Following KPro

Placement of the PMMA optic and the 8.5 mm backplate invalidates both central and peripheral applanation values. Tono-Pen measurements at the limbus may provide a rough assessment of the IOP (particularly when compared with measurements from the other eye taken at a corresponding limbal location), but the readings obtained are highly variable. IOP estimates using globe palpation is the most commonly employed method of estimating IOP in patients post-KPro implantation. Digital palpation of the globe should be performed with the patient gently looking down and the fingers places above the tarsal plate. Finger tension has been demonstrated to be fairly accurate at detecting IOPs of 30 mm Hg or more particularly when performed by experienced observers [65].

### 5.4. Management of Glaucoma Following KPro

The management of glaucoma following KPro is usually surgical. Some surgeons consider placing a GDD implant in all patients undergoing KPro surgery. A meticulous preoperative evaluation of the angles and optic disc, either clinically or with help of ultrasound, is mandatory in order to assess postoperative development or progression of glaucoma. GDD implantation is usually done at the time of KPro surgery in cases with mild optic nerve damage. Dohlman et al. [66] studied the connection of the Ahmed shunts to distant epithelialized cavities (lacrimal sac, ethmoidal sinuses, maxillary sinus, and the lower lid fornix). The incidence of severe infection was very low, in fact comparable to that after standard trabeculectomy.

Patients with uncontrolled IOP or advanced optic nerve damage should ideally have glaucoma surgery in the form of GDD implantation or CPC 3–6 months prior to KPro surgery to know how many medications will be required after KPro surgery.

Only patients with open angles and normal pre-KPro pressures off medications, without anticipated manipulation of the lens or iris, should proceed to KPro placement without glaucoma surgical intervention [65].

CPC can be performed at the time of or subsequent to KPro placement. Success has been reported with both endoscopic and transscleral methods [63, 64, 67, 68]. Multiple applications may be required over time.

## 6. Glaucoma and Corneal Refractive Surgery

Though refractive surgery does not strictly qualify as a corneal transplantation, the structure of the cornea is altered dramatically and deserves brief comment in this paper.

### 6.1. Etiology of Glaucoma Following Corneal Refractive Surgery

During LASIK surgery, large intraocular spikes can occur during corneal flap construction, which might acutely damage the optic nerve or cause retinal vascular occlusions [69–72]. The IOP spikes may be less severe with femtosecond laser-assisted LASIK. In addition, refractive surgery patients are often moderate-to-high myopic, who may have a higher predisposition to the development of primary open angle glaucoma (POAG) [73, 74], pigmentary glaucoma [74, 75], and steroid-induced glaucoma [76, 77].

### 6.2. IOP Measurement Following Corneal Refractive Surgery

The measurement of IOP after corneal refractive surgery may not be accurate because of changes in central corneal thickness. Corneal ablative procedures decrease CCT and alter structural biomechanical properties of the cornea resulting in spuriously low IOP measurement.

Standard GAT pressures are altered after refractive surgery due to biomechanical changes in cornea [78–81]. No reliable nomograms to estimate the effects of refractive surgery on corneal dynamics and IOP exist. Tono-Pen and pneumotonometry are less affected by refractive surgery compared to GAT [81–84]. DCT or Ocular response analyzer (ORA) may provide more reproducible data when comparing postoperative IOP with baseline [85].

## 7. Glaucoma and Corneal Collagen Cross-Linkage

Corneal stiffening following corneal CXL could potentially alter corneal biomechanics and the measurement of IOP, but this effect has not been studied extensively. Kymionis et al. [86] reported a statistically significant increase in GAT measured IOP 6 months and 12 months after CXL (both *P* < 0.001). The mean IOP was 9.95 mm Hg ± 3.01 before CXL, 11.40 ± 2.89 mm Hg at 6 months, and 11.35 ± 3.38 mm Hg at 12 months. This slight change was probably caused by an increase in corneal rigidity.

## 8. Conclusion

Glaucoma is a well-established complication following corneal transplant procedures. In addition, corneal surgeries that either increase or decrease the corneal thickness represent a challenge for IOP measurement and establishment of a diagnosis of glaucoma. It is mandatory that the intraocular pressure should be monitored on a regular basis after corneal transplantation procedures and aggressively treated if found to be high. Any patient with preexisting glaucoma must be carefully evaluated prior to the corneal transplants. It is equally important, when possible, to obtain other tests such as visual field testing and optic disc imaging to monitor progression of glaucoma. The lack of prospective studies adds to the difficulty of determining the true incidence, risk factors, and standard protocols for management. Multicentre, prospective studies may help establish better protocols for followup and management of patients with elevated IOP following corneal procedures to avoid the devastating outcomes of glaucoma.
